# Covariate adjustment of spirometric and smoking phenotypes: The potential of neural network models

**DOI:** 10.1371/journal.pone.0266752

**Published:** 2022-05-11

**Authors:** Kirsten Voorhies, Ruofan Bie, John E. Hokanson, Scott T. Weiss, Ann Chen Wu, Julian Hecker, Georg Hahn, Dawn L. Demeo, Edwin Silverman, Michael H. Cho, Christoph Lange, Sharon M. Lutz

**Affiliations:** 1 Department of Population Medicine, PRecisiOn Medicine Translational Research (PROMoTeR) Center, Harvard Pilgrim Health Care and Harvard Medical School, Boston, MA, United States of America; 2 Department of Biostatistics, T.H. Chan School of Public Health, Harvard University, Boston, MA, United States of America; 3 Department of Epidemiology, University of Colorado Anschutz, Aurora, CO, United States of America; 4 Channing Division of Network Medicine, Brigham and Women’s Hospital, Harvard Medical School, Boston, MA, United States of America; 5 Division of General Pediatrics, Department of Pediatrics, Children’s Hospital, Boston, MA, United States of America; University of Nevada Reno, UNITED STATES

## Abstract

To increase power and minimize bias in statistical analyses, quantitative outcomes are often adjusted for precision and confounding variables using standard regression approaches. The outcome is modeled as a linear function of the precision variables and confounders; however, for many complex phenotypes, the assumptions of the linear regression models are not always met. As an alternative, we used neural networks for the modeling of complex phenotypes and covariate adjustments. We compared the prediction accuracy of the neural network models to that of classical approaches based on linear regression. Using data from the UK Biobank, COPDGene study, and Childhood Asthma Management Program (CAMP), we examined the features of neural networks in this context and compared them with traditional regression approaches for prediction of three outcomes: forced expiratory volume in one second (FEV_1_), age at smoking cessation, and log transformation of age at smoking cessation (due to age at smoking cessation being right-skewed). We used mean squared error to compare neural network and regression models, and found the models performed similarly unless the observed distribution of the phenotype was skewed, in which case the neural network had smaller mean squared error. Our results suggest neural network models have an advantage over standard regression approaches when the phenotypic distribution is skewed. However, when the distribution is not skewed, the approaches performed similarly. Our findings are relevant to studies that analyze phenotypes that are skewed by nature or where the phenotype of interest is skewed as a result of the ascertainment condition.

## Introduction

In epidemiological studies of respiratory diseases and smoking phenotypes, prediction models are often fit using standard linear regression. However, a linear regression model assumes there is a linear relationship between the mean of the phenotype and the covariates. While this might be a reasonable assumption for some parts of the phenotypic range, it is questionable whether linearity holds in the tails of the distribution, especially when diseased populations are analyzed and the majority of study subjects have phenotypic values that are in the tails of the distribution.

Neural networks, a well-developed deep learning approach [1], can describe non-linear relationships between predictors and outcomes and are often able to achieve more accurate prediction than those based on linear regression, making them potentially useful for predicting complex respiratory phenotypes and smoking traits. Two important questions in epidemiology are hypothesis testing and prediction. Hypothesis testing focuses on whether a variable X is associated with an outcome Y, and whether other variables are confounders or precision variables. Prediction focuses on improving predictive accuracy by including all covariates with appropriate forms that improve the prediction and excluding covariates that do not improve the prediction accuracy of the model. Machine learning methods can provide a tool to investigate covariates to include and forms of covariates to be used.

Previous work found machine learning methods can predict smoking cessation and forced expiratory volume in one second (FEV_1_), a spirometric measure used to determine COPD severity [2–4]. In particular, radial basis neural network predicted FEV_1_ using spirometry data [5], and spirometry and demographic data [6], and the predicted and actual FEV_1_ values were highly correlated. However, prediction accuracy was better for normal rather than restrictive or obstructive diseased condition [5, 6]. Therefore, there is evidence machine learning and deep learning methods can be used to predict these outcomes, and they can offer advantages over other models in some circumstances.

We evaluated the prediction properties of neural network models as compared to standard regression models. We used data from the UK Biobank [7], the COPDGene study [8], and the Childhood Asthma Management Program (CAMP) [9] to assess the performance of both approaches by comparing the test mean squared error (MSE) of each approach and each data set. For each study we predicted FEV_1_, and using the UK Biobank and COPDGene study, we also predicted age at smoking cessation and log age at smoking cessation.

## Methodology

For the linear regression model, let *y*_*i*_ denote the outcome, where *i* is the *i*^*th*^ study subject. Let *k* be the number of covariates $x_{1_{i}},‥,x_{k_{i}}$. To simplify, we denoted the covariate matrix as *X* and **x_i_** is the *i*^*th*^ subject in the matrix. We assumed a linear relationship and used the training set to estimate parameters in the following equation:

$$\begin{array}{c} E(y_{i}|x_{i})=\beta_{0}+\beta_{1}x_{1_{i}}+\beta_{2}x_{2_{i}}+\cdots +\beta_{k}x_{k_{i}} \end{array}$$ (1)

Neural networks are made up of layers of neurons, and the number of neurons and layers can vary depending on the data. The input layer of the neural network has a neuron for each of the predictors from the data set being used, any hidden layers each have the number of neurons specified by the user, and the output layer has one neuron when predicting a single continuous outcome [10]. The number of hidden layers and neurons for each hidden layer are typically determined by trial and error. For this study, we used two hidden layers. Each neuron has an associated weight, and the sum of the neurons multiplied by their weights is input into an activation function, which outputs to the next layer. Activation functions are specified for each hidden layer and the output layer.

For the neural network model, suppose there are *p* layers in the model denoted *L*_1_, *L*_2_, ⋯, *L*_*p*_. For the *i*^*th*^ layer, there are *n*_*i*_ neurons, each neuron is denoted $N_{1_{i}},N_{2_{i}},\cdots ,N_{n_{i}}$, and the layer uses activation function *ϕ*_*i*_. The activation function works as a link function and converts the input signal to the output signal on a node. For example, a linear activation function is *g*(*x*) = *x*, which is commonly used in linear regression models, while a non-linear activation function, such as sigmoid function $g\left(x\right)=\frac{1}{1+e^{-x}}$, can be used in a neural network model. Karlik and Olgac (2011), and Sibi et al. (2013) provide more details and comparison of activation functions [11, 12]. The following equation is used for calculating $N_{j_{i+1}}$, the *j*^*th*^ neuron in the *i*^*th*^ + 1 layer:

$$\begin{array}{c} N_{j_{i+1}}=\phi_{i}(\sum_{k_{i}=1}^{n_{i}}w_{k_{i}}N_{k_{i}}) \end{array}$$ (2)

where $w_{k_{i}}$ is the weight for the *k*^*th*^ neuron in the *i*^*th*^ layer.

To evaluate prediction accuracy, we applied the trained models on the test data to predict FEV_1_, age at smoking cessation, and log age at smoking cessation. We used data from the UK Biobank, COPDGene study, and CAMP. The UK Biobank is a large prospective study [7], COPDGene is a study of smokers in which participants were enrolled based on COPD affection status [8], and CAMP is a study of children with asthma [9]. For the UK Biobank and CAMP, we included subjects of European ancestry. For the COPDGene study, we included African American and non-Hispanic white participants in separate models. Ethnicity was based on self-report. To predict FEV_1_, the models included age, sex, BMI, centered height, and squared centered height as covariates. According to previous literature, these are common factors that may be associated with FEV_1_ [13, 14]. Height and height squared were centered to reduce correlation between these two covariates. We considered two samples for prediction of FEV_1_ using the UK Biobank data, one sample which included all subjects, and another sample which only included a subset of subjects with the lowest 20% of FEV_1_ measurements to create ascertainment bias. To predict age at smoking cessation and log age at smoking cessation, we included former smokers, and the models included age, sex, age started smoking, education (attended college or university), pack years of cigarettes, and smoker in household. Age at smoking cessation was measured in the UK Biobank by asking participants who had stopped smoking “At what age did you give up?”, and in the COPDGene study by asking participants “How old were you when you completely stopped smoking?”. Characteristics of subjects are shown in Table 1.

**Table 1:** Characteristics of subjects from the UK Biobank, COPDGene, and CAMP data. For continuous variables, we give the mean and standard deviation (i.e. mean (sd)). Sample 1 is for FEV_1_ as the outcome. Sample 2 is for age at smoking cessation as the outcome and includes former smokers. Sample 3 is for FEV_1_ as the outcome for the subjects with the lowest 20% of FEV_1_.

	UK Biobank	COPDGene: non-Hispanic white	COPDGene: African American	CAMP
Sample 1, *n*	151,879	6,764	3,365	698
FEV_1_	2.77 (0.75)	2.22 (0.95)	2.29 (0.86)	1.83 (0.50)
Sex (male), *n* (%)	88,406 (58.21)	3,553 (52.53)	1,856 (55.16)	408 (58.45)
Age, years	56.25 (7.98)	62.02 (8.86)	54.66 (7.21)	8.85 (2.13)
BMI	27.52 (4.86)	28.68 (6.05)	29.07 (6.66)	17.78 (3.05)
Height, cm	167.84 (9.08)	169.74 (9.46)	171.01 (9.67)	132.84 (13.84)
Sample 2, *n*	21,142	4,104	673	-
Smoking cessation, age in years	37.03 (10.33)	50.92 (11.03)	51.51 (9.66)	-
Education (college or university), *n* (%)	9,201 (43.52)	3,039 (74.05)	341 (50.67)	-
Pack years	18.09 (14.46)	46.71 (26.96)	38.51 (22.29)	-
Smoker in household, *n* (%)	2,338 (11.06)	3,268 (79.63)	521 (77.41)	-
Age started smoking, years	17.43 (3.18)	16.95 (3.85)	17.13 (4.97)	-
Sample 3, *n*	29,805	-	-	-
FEV_1_	1.81 (0.28)	-	-	-
Sex (male), *n* (%)	26,078 (87.50)	-	-	-
Age, years	61.00 (6.27)	-	-	-
BMI	28.26 (5.53)	-	-	-
Height, cm	160.77 (7.06)	-	-	-

We randomly selected 1,000 subsets of the data sets to compare the mean test MSE for the neural network and linear regression models where 50%, 25%, or 10% of the sampled data was used as the test data. Each model was trained using the other 50%, 75%, or 90% of the sampled data. Activation functions used and number of neurons for each model are included in Table 2, and the architecture of the models is shown in S4 and S5 Figs in S1 Appendix. As seen in Table 2, we used sigmoid functions for FEV_1_, hard sigmoid and rectified linear unit (RELU) for smoking cessation, and sigmoid functions for log smoking cessation. Analyses were done in R, and we used the package ‘Keras’ for the neural network analyses [15], and the package ‘caret’ for partitioning the data into the test and training data sets [16].

**Table 2:** Best neural network model features for predicting the different outcomes, determined by testing different combinations of activation functions, number of layers, and number of neurons per layer for each data set.

Outcome	First Hidden Layer		Second Hidden Layer	
	Activation Function	Neurons	Activation Function	Neurons
FEV_1_	Sigmoid	64	Sigmoid	16
Smoking Cessation	Hard Sigmoid	64	RELU	32
Log Smoking Cessation	Sigmoid	64	Sigmoid	32

## Data analysis

We applied the neural network models and linear regression models to predict FEV_1_ using the UK Biobank data among subjects of European ancestry (N = 151,879), a subset of the UK Biobank data among subjects of European ancestry limited to subjects with the lowest 20% of FEV_1_ measurements (N = 29,805), COPDGene study data among non-Hispanic white subjects (N = 6,764), COPDGene study data among African American subjects (N = 3,365), and CAMP data among subjects of European ancestry (N = 698), and to predict age at smoking cessation and log age at smoking cessation using the UK Biobank data among subjects of European ancestry (N = 21,142), COPDGene study data among non-Hispanic white subjects (N = 4,104), and COPDGene study data among African American subjects (N = 673). Note that all data is from phase 1 of the COPDGene study.

**Fig 1:**
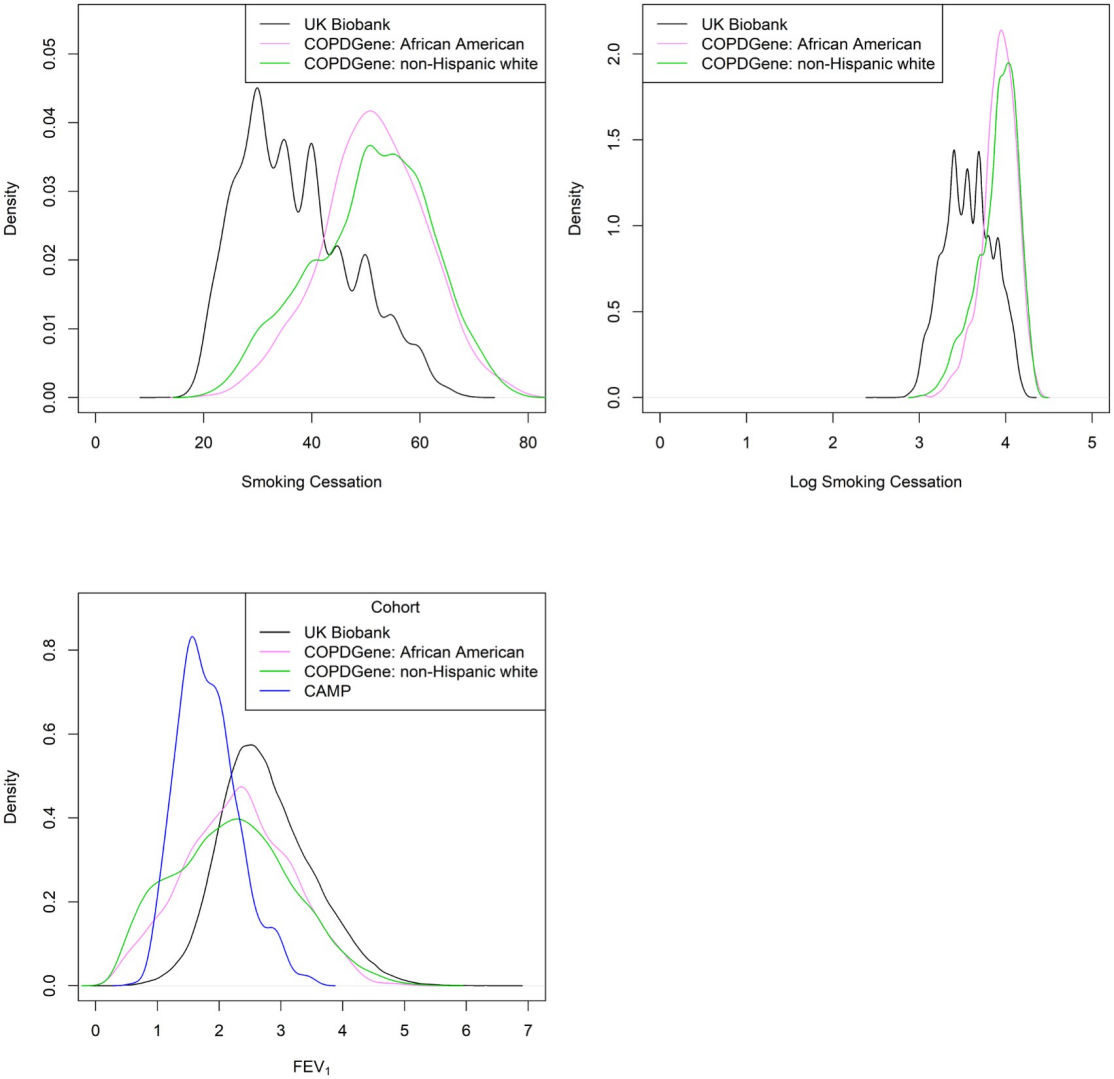
The plot in the top left shows the density plot of smoking cessation (age). The plot in the top right shows the density plot of log smoking cessation (age). The plot in the bottom left shows the density plot of FEV_1_.

Density plots of the outcomes revealed FEV_1_ was normally distributed, but age at smoking cessation was right-skewed and could benefit from a log transformation. Density plots of the distributions are shown in Fig 1.

We evaluated the predictive performance of the models by calculating the test MSE for each model. For every data set, we separated 50%, 75%, or 90% of the sample as the training data, and the remaining 50%, 25%, or 10% was used as the test data. Using the training data, the neural network models and the linear regression models were fit, and then these models predicted the outcome *y* for the test data.

## Results

**Fig 2:**
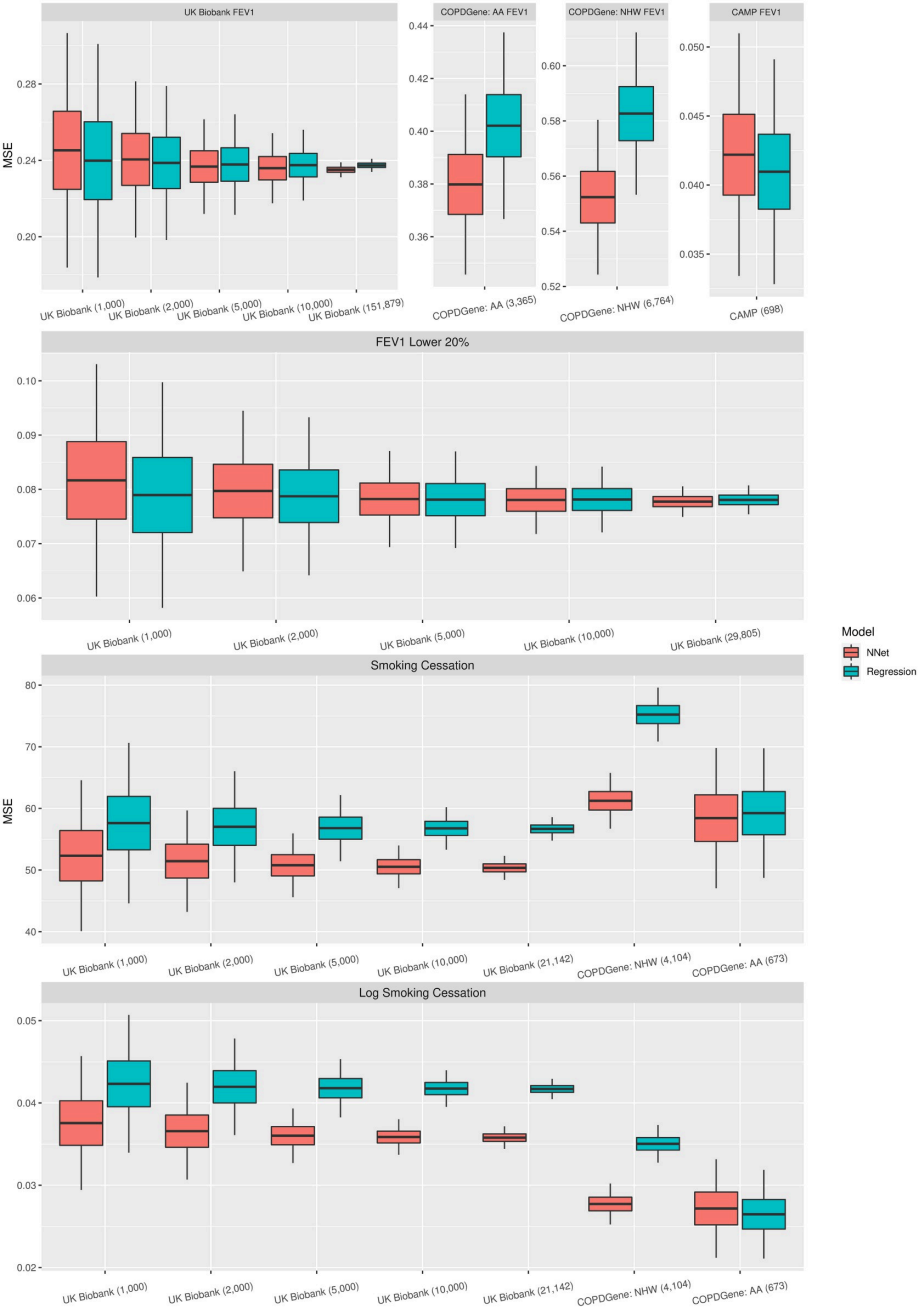
This figure includes box plots of the MSE for the different studies and outcomes when 50% of the data was used to train the models.

The MSE of the test data for the linear regression and neural network models for the different data sets, sample sizes, and different proportions of data used for the test and training data are shown in Fig 2 and in the S1-S3 Figs and S1-S3 Tables in S1 Appendix. As we decreased the test data size, the standard error of the MSE increased, while the MSE was either similar for all three test data size percentages (50%, 25%, and 10%) or decreased as the percent test data decreased.

For the prediction of FEV_1_ for all subjects, the MSE was similar for neural network and linear regression across all data sets, sample sizes, and proportions of test data used except for CAMP, where the MSE for linear regression was smaller than for neural network. For the prediction of FEV_1_ for UK Biobank subjects with the lowest 20% FEV_1_ measurements, the MSE was similar for neural network and linear regression for all sample sizes and proportions of test data used. For the prediction of age at smoking cessation, the MSE was smaller for the neural network models for all data sets, sample sizes, and proportions of test data used, and thus the neural network models showed an advantage in prediction over linear regression. The neural network models showed the largest advantage over the linear regression models when examining the COPDGene study among non-Hispanic white subjects. For the COPDGene study among African American subjects, the neural network models still had a smaller MSE when predicting age at smoking cessation, however, the difference was less than in the other data sets. For the prediction of log age at smoking cessation, the MSE was smaller for neural network than linear regression across all data sets, sample sizes, and proportions of test data used except for the COPDGene study among African American subjects, for which linear regression had a slightly smaller MSE when 50% of the data was used for testing. The neural network models had the largest advantage over the linear regression models when examining the COPDGene study among non-Hispanic white subjects.

## Discussion

We used multiple permutations of subsets of the data to compare the prediction accuracy of linear regression and neural networks for three continuous outcomes, FEV_1_, age at smoking cessation, and log age at smoking cessation. The linear regression and neural network models had similar MSE when the outcome was normally distributed (FEV_1_), but the neural network model generally had smaller MSE than the linear regression when the outcome was not normally distributed (age at smoking cessation) or had been transformed (log age at smoking cessation). This difference was largest for the COPDGene study among non-Hispanic white subjects, and smallest for the COPDGene study among African American subjects. The subset of the COPDGene study among African American subjects had the smallest sample size for age at smoking cessation, which could be a reason we saw less of a difference in MSE between the linear regression model and neural network model for age at smoking cessation, and potentially could explain why the MSE was smaller for the linear regression when predicting log age at smoking cessation using 50% of the data to test. While neural network had better prediction accuracy in some scenarios, the interpretability of regression is superior to neural networks as the coefficients in the regression model have a straightforward interpretation.

Previous research found success in using backpropagation neural network to classify current and former smokers, with classification performance better than chance. However, compared to a logistic regression model on the same data, they found prediction was not improved when using the backpropagation neural network instead of the logistic regression [17]. Successful prediction of FEV_1_ has also been found using neural networks previously, with one study aiming to see if neural network models could predict FEV_1_ better than previously published predictions that used multiple regression analysis. Using the same sample of elderly adults as the previous model, the neural network predictions were found to correlate better to the FEV_1_ values than the predictions made by the regression analysis [18].

There were some limitations of our analysis. While we considered continuous outcomes, we did not consider binary outcomes. Additionally, while the neural network models generally had lower MSE than the regression models when the phenotypic distribution was skewed, we do not know if this is specific to the data we used or a general property of neural networks. Also, it is important to note that our observations are based on only a few predictors and three data sets. We used MSE of the test data to measure and compare prediction accuracy; however, other metrics could be used to measure model fit.

While we focused on covariate adjustment of spirometric and smoking phenotypes, future research could examine if the covariate adjustment using neural networks improves the performance of genome wide association studies (GWAS) for rare or common variants. Reducing variability in the outcome should increase power for GWAS, and it is not clear if using neural networks to improve covariate adjustment for spirometric and smoking phenotypes could lead to novel variants. While we considered outcomes related to smoking and lung function, it could be worth considering additional health outcomes in the future.

To summarize, we compared regression and neural network analyses based on test MSE, and found for our outcomes there were scenarios where the regression and neural network models performed similarly well. However, when the phenotypic distribution was skewed in our data, the neural network model had a lower average test MSE in our analyses.

## Supporting information

S1 Appendix Additional tables, plots, and COPDGene study information. (PDF)
