# Supplementary material for: Covariate adjustment of spirometric and smoking phenotypes: The potential of neural network models
Source: PLoS One. 2022 May 11;17(5):e0266752. doi: 10.1371/journal.pone.0266752 (PMC9094505; doi:10.1371/journal.pone.0266752)
Supplement: S1 Appendix — (PDF) [file pone.0266752.s001.pdf]

Supplementary materials for "Covariate adjustment of spirometric and smoking phenotypes: The potential of neural network models"

Table S1: Test MSE for neural network and linear regression models across different sample sizes and outcomes with 50% of the sample used to train the models. The p-value column shows the p-value of the test of difference in MSEs; caution is needed when interpreting this p-value.

Outcome	Dataset	Sample Size	Linear Regression	Neural Network	p-value
FEV_1	UK Biobank	1,000	2.40e-1 (2.04e-2)	2.45e-1 (2.05e-2)	3.12e-9
		2,000	2.39e-1 (1.34e-2)	2.40e-1 (1.36e-2)	2.48e-3
		5,000	2.38e-1 (8.76e-3)	2.37e-1 (8.25e-3)	4.67e-3
		10,000	2.37e-1 (6.15e-3)	2.36e-1 (6.11e-3)	6.30e-9
		151,879	2.37e-1 (1.13e-3)	2.35e-1 (1.29e-3)	5.95e-286
	COPDGene: non-Hispanic white	6,764	5.83e-1 (9.80e-3)	5.52e-1 (9.34e-3)	$p<1e-99$
	COPDGene:African American	3,365	4.02e-1 (1.18e-2)	3.80e-1 (1.14e-2)	2.52e-286
	CAMP	698	4.10e-2 (2.71e-3)	4.22e-2 (2.93e-3)	5.95e-22
FEV_1 Lowest 20%	UK Biobank	1,000	7.90e-2 (6.92e-3)	8.17e-2 (7.13e-3)	1.78e-17
		2,000	7.87e-2 (4.85e-3)	7.97e-2 (4.93e-3)	9.17e-6
		5,000	7.81e-2 (2.96e-3)	7.82e-2 (2.95e-3)	0.39
		10,000	7.81e-2 (2.02e-3)	7.81e-2 (2.08e-3)	0.31
		29,805	7.81e-2 (8.83e-4)	7.78e-2 (9.31e-4)	7.91e-15
Smoking Cessation	UK Biobank	1,000	57.79 (4.46)	52.55 (4.06)	1.23e-146
		2,000	56.98 (3.02)	51.48 (2.89)	6.08e-289
		5,000	56.66 (1.77)	50.71 (1.67)	$p<1e-99$
		10,000	56.60 (1.14)	50.48 (1.06)	$p<1e-99$
		21,142	56.51 (0.63)	50.35 (0.62)	$p<1e-99$
	COPDGene: non-Hispanic white	4,104	75.22 (1.46)	61.24 (1.50)	$p<1e-99$
	COPDGene: African American	673	59.24 (3.50)	58.42 (3.79)	6.72e-7
Log Smoking Cessation	UK Biobank	1,000	4.23e-2 (2.79e-3)	3.76e-2 (2.71e-3)	8.08e-246
		2,000	4.20e-2 (1.96e-3)	3.66e-2 (1.96e-3)	$p<1e-99$
		5,000	4.18e-2 (1.18e-3)	3.60e-2 (1.10e-3)	$p<1e-99$
		10,000	4.18e-2 (7.39e-4)	3.59e-2 (7.17e-4)	$p<1e-99$
		21,142	4.17e-2 (4.08e-4)	3.58e-2 (4.55e-4)	$p<1e-99$
	COPDGene: non-Hispanic white	4,104	3.50e-2 (7.61e-4)	2.77e-2 (8.27e-4)	$p<1e-99$
	COPDGene:African American	673	2.65e-2 (1.79e-3)	2.72e-2 (1.99e-3)	2.32e-16

Figure S1: This figure includes box plots of the test MSE for the different data and outcomes when 50% of the data was used to train the models.

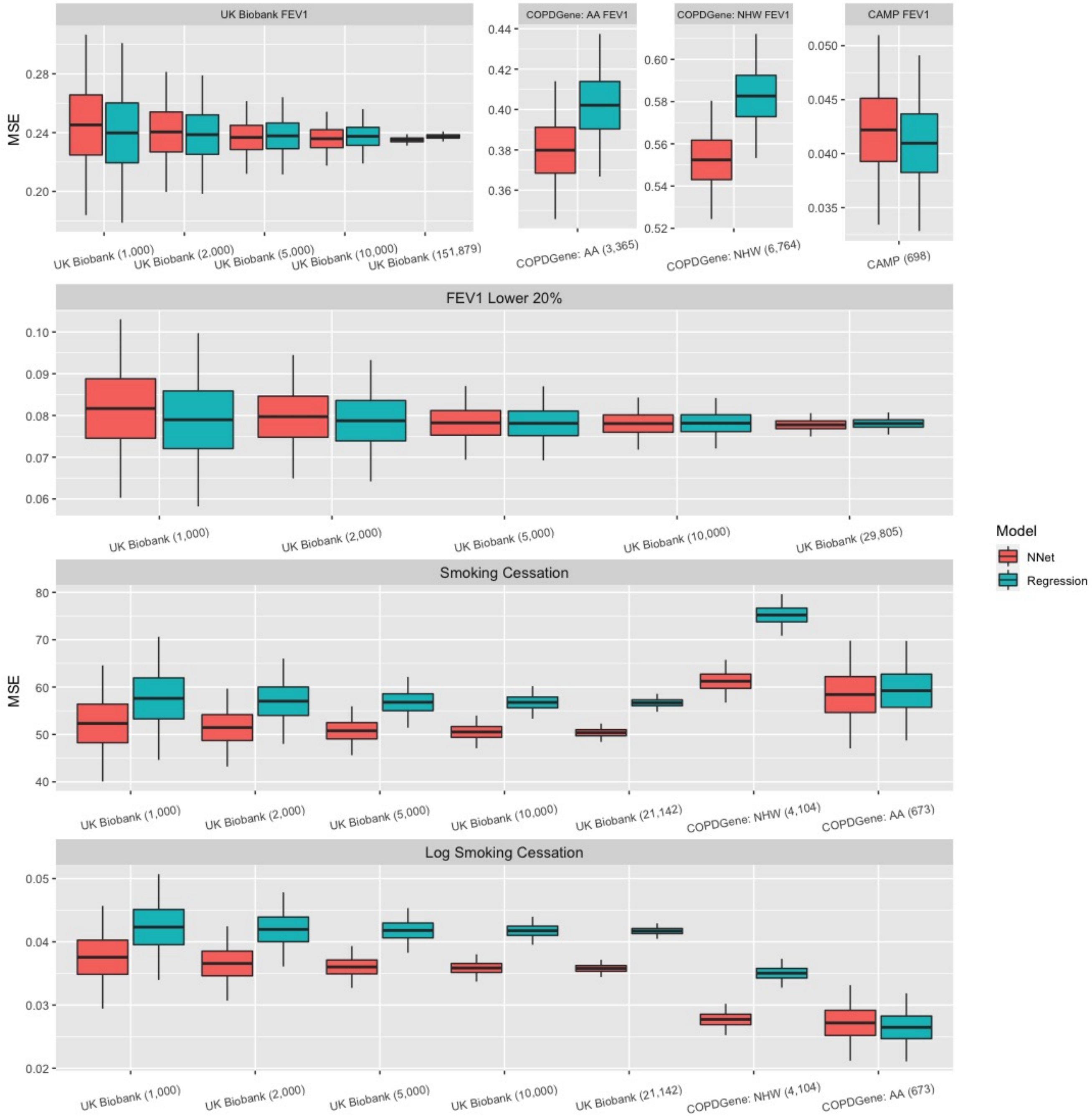

Table S2: Test MSE for neural network and linear regression models across different sample sizes and outcomes with 75% of the sample used to train the models. The p-value column shows the p-value of the test of difference in MSEs; caution is needed when interpreting this p-value.

Outcome	Dataset	Sample Size	Linear Regression	Neural Network	p-value
FEV_1	UK Biobank	1,000	2.39e-1 (2.77e-2)	2.42e-1 (2.83e-2)	2.13e-2
		2,000	2.38e-1 (1.85e-2)	2.39e-1 (2.00e-2)	6.34e-2
		5,000	2.37e-1 (1.23e-2)	2.37e-1 (1.24e-2)	0.14
		10,000	2.37e-1 (8.64e-3)	2.36e-1 (8.69e-3)	2.62e-4
		151,879	2.37e-1 (1.95e-3)	2.35e-1 (2.01e-3)	2.68e-135
	COPDGene: non-Hispanic white	6,764	5.82e-1 (1.64e-2)	5.50e-1 (1.63e-2)	2.65e-283
	COPDGene: African American	3,365	4.01e-1 (1.98e-2)	3.78e-1(1.84e-2)	4.36e-129
	CAMP	698	4.06e-2 (4.57e-3)	4.13e-2 (4.47e-3)	8.94e-4
FEV_1 Lowest 20%	UK Biobank	1,000	7.87e-2 (9.75e-3)	8.00e-2 (9.96e-3)	1.99e-3
		2,000	7.86e-2 (6.93e-3)	7.89e-2 (6.80e-3)	0.33
		5,000	7.80e-2 (4.34e-3)	7.81e-2 (4.33e-3)	0.86
		10,000	7.82e-2 (2.98e-3)	7.79e-2 (3.00e-3)	1.47e-2
		29,805	7.80e-2 (1.51e-3)	7.77e-2 (1.57e-3)	5.64e-6
Smoking Cessation	UK Biobank	1,000	57.37 (6.12)	51.74 (5.72)	1.06e-90
		2,000	57.08 (4.28)	51.03 (3.86)	1.75e-192
		5,000	56.59 (2.56)	50.61 (2.48)	p<1e-99
		10,000	56.68 (1.78)	50.40 (1.71)	p<1e-99
		21,142	56.69 (1.15)	50.30 (1.05)	p<1e-99
	COPDGene: non-Hispanic white	4,104	75.05 (2.46)	60.93 (2.55)	p<1e-99
	COPDGene: African American	673	59.17 (6.16)	57.09 (6.17)	5.73e-14
Log Smoking Cessation	UK Biobank	1,000	4.21e-2 (3.97e-3)	3.68e-2 (3.63e-3)	2.50e-176
		2,000	4.20e-2 (2.74e-3)	3.62e-2 (2.54e-3)	p<1e-99
		5,000	4.16e-2 (1.66e-3)	3.60e-2 (1.56e-3)	p<1e-99
		10,000	4.17e-2 (1.16e-3)	3.58e-2 (1.09e-3)	p<1e-99
		21,142	4.17e-2 (7.49e-4)	3.58e-2 (6.84e-4)	p<1e-99
	COPDGene: non-Hispanic white	4,104	3.49e-2 (1.30e-3)	2.76e-2 (1.27e-3)	p<1e-99
	COPDGene: African American	673	2.65e-2 (3.21e-3)	2.64e-2 (3.19e-3)	0.49

Figure S2: This figure includes box plots of the test MSE for the different data and outcomes when 75% of the data was used to train the models.

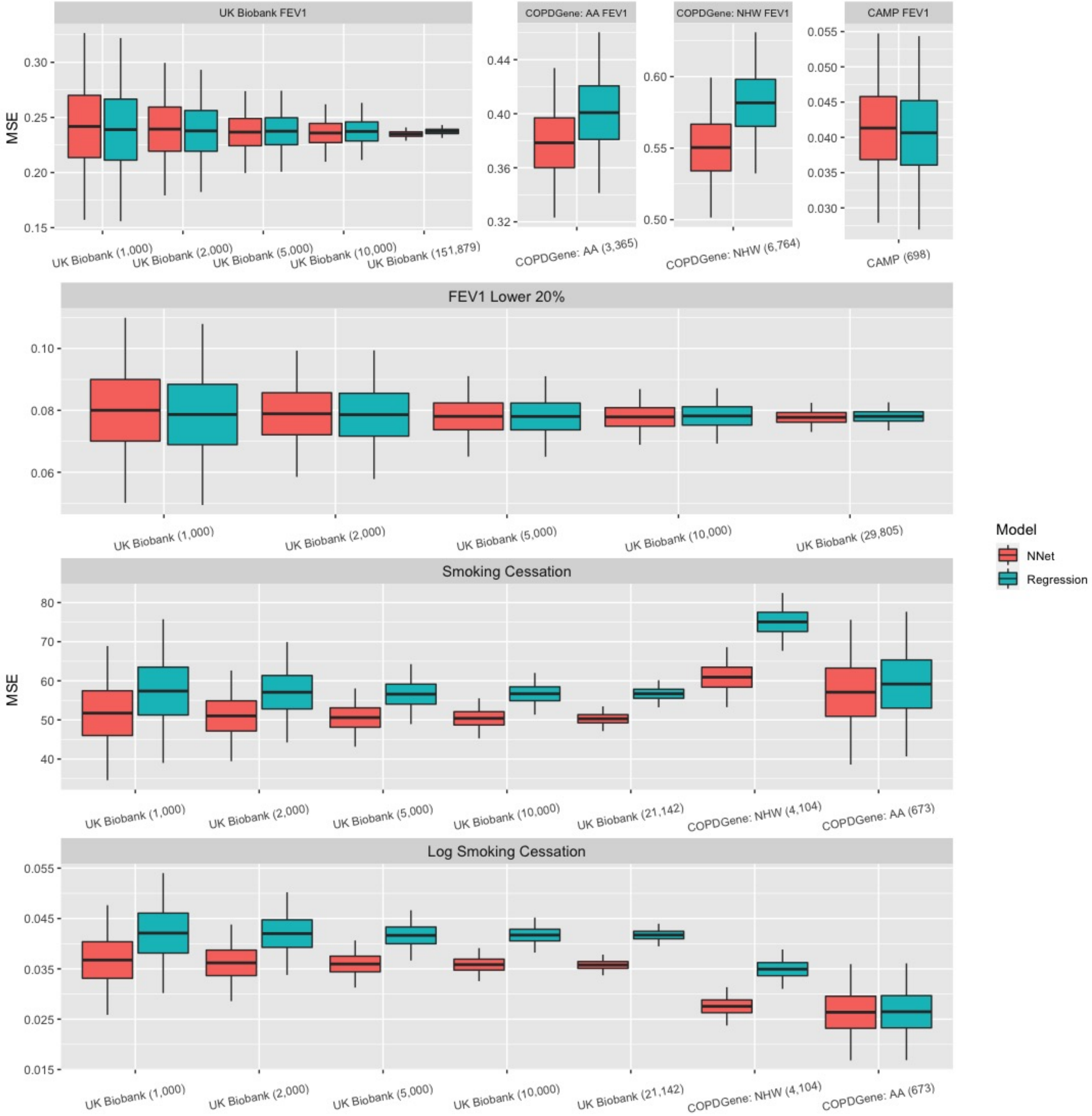

Table S3: Test MSE for neural network and linear regression models across different sample sizes for different outcomes with 90% of the sample used to train the models. CAMP was excluded from prediction of FEV_1, and the COPDGene study among African American subjects was excluded from prediction of smoking cessation (age) and log smoking cessation (age) due to the small sample sizes. The p-value column shows the p-value of the test of difference in MSEs; caution is needed when interpreting this p-value.

Outcome	Dataset	Sample Size	Linear Regression	Neural Network	p-value
FEV_1	UK Biobank	1,000	2.41e-1 (4.46e-2)	2.42e-1 (4.31e-2)	0.51
		2,000	2.38e-1 (2.97e-2)	2.37e-1 (3.17e-2)	0.58
		5,000	2.38e-1 (1.90e-2)	2.37e-1 (2.01e-2)	0.29
		10,000	2.37e-1 (1.41e-2)	2.35e-1 (1.37e-2)	1.24e-4
		151,879	2.37e-1 (3.36e-3)	2.35e-1 (3.34e-3)	8.31e-57
	COPDGene: non-Hispanic white	6,764	5.81e-1 (2.91e-2)	5.51e-1 (2.89e-2)	7.71e-104
	COPDGene: African American	3,365	4.02e-1 (3.18e-2)	3.78e-1 (3.34e-2)	1.61e-56
	CAMP	698	NA	NA	NA
FEV_1 Lowest 20%	UK Biobank	1,000	7.81e-2 (1.53e-2)	8.00e-2 (1.58e-2)	7.12e-3
		2,000	7.85e-2 (1.07e-2)	7.87e-2 (1.06e-2)	0.66
		5,000	7.81e-2 (7.08e-3)	7.83e-2 (6.95e-3)	0.52
		10,000	7.83e-2 (4.87e-3)	7.77e-2 (4.90e-3)	8.77e-3
		29,805	7.80e-2 (2.77e-3)	7.77e-2 (2.76e-3)	3.85e-3
Smoking Cessation	UK Biobank	1,000	57.58 (9.65)	51.40 (8.95)	2.80e-47
		2,000	57.17 (6.79)	51.33 (6.53)	2.15e-78
		5,000	56.58 (4.21)	50.59 (4.02)	1.89e-186
		10,000	56.65 (2.95)	50.40 (2.58)	p<1e-99
		21,142	56.73 (1.90)	50.24 (1.85)	p<1e-99
	COPDGene: non-Hispanic white	4,104	75.03 (4.29)	60.87 (4.37)	p<1e-99
	COPDGene: African American	673	NA	NA	NA
Log Smoking Cessation	UK Biobank	1,000	4.23e-2 (6.14e-3)	3.64e-2 (5.75e-3)	3.22e-96
		2,000	4.21e-2 (4.38e-3)	3.60e-2 (4.04e-3)	7.85e-182
		5,000	4.16e-2 (2.70e-3)	3.57e-2 (2.60e-3)	p<1e-99
		10,000	4.17e-2 (1.90e-3)	3.58e-2 (1.73e-3)	p<1e-99
		21,142	4.17e-2 (1.23e-3)	3.58e-2 (1.23e-3)	p<1e-99
	COPDGene: non-Hispanic white	4,104	3.49e-2 (2.29e-3)	2.75e-2 (2.09e-3)	p<1e-99
	COPDGene: African American	673	NA	NA	NA

Figure S3: This figure includes box plots of test MSE for the different data and outcomes when 90% of the data was used to train the models. CAMP was excluded from prediction of FEV_1, and the COPDGene study among African American subjects was excluded from prediction of smoking cessation (age) and log smoking cessation (age) due to the small sample sizes.

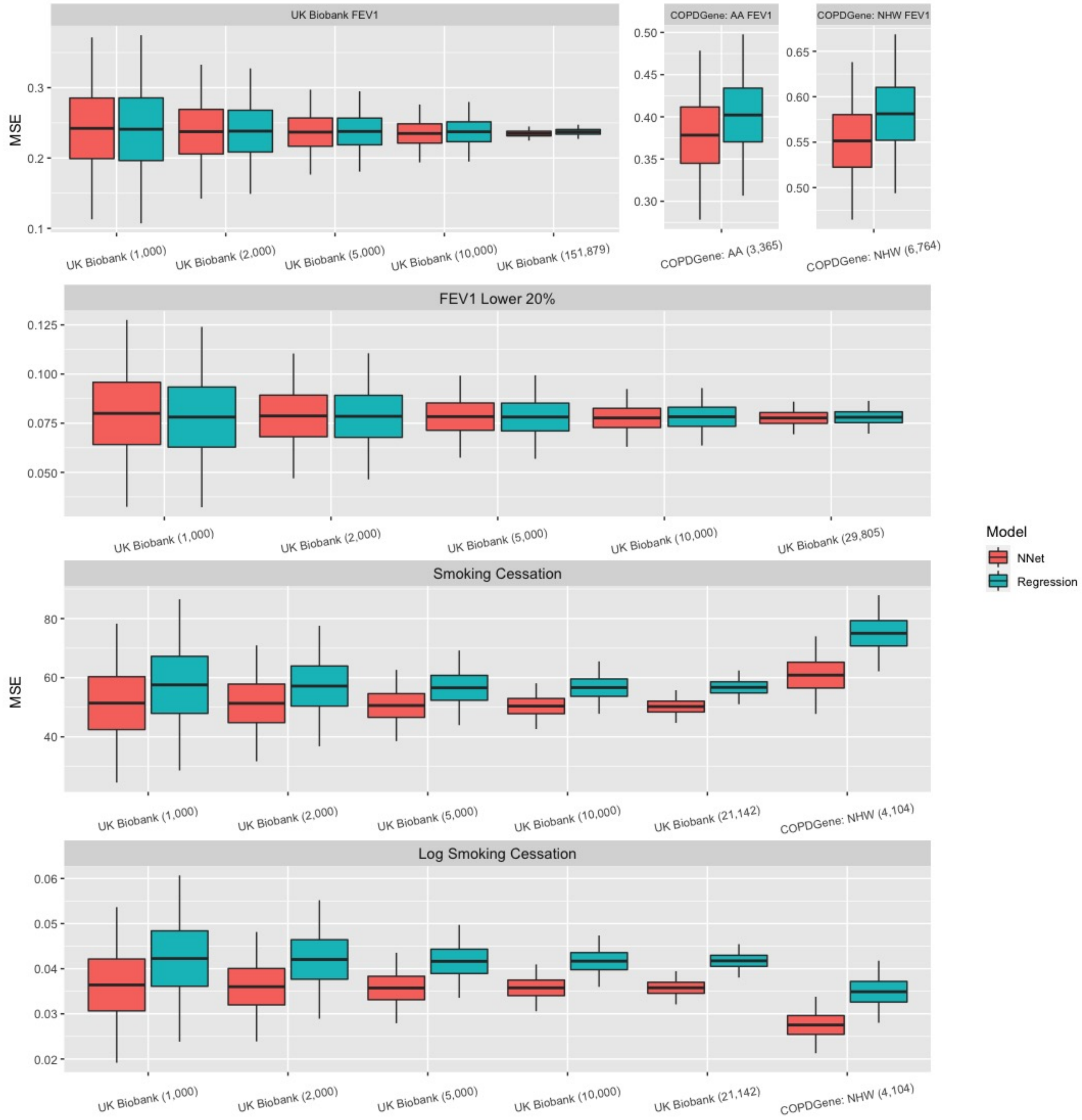

Figure S4: Architecture of neural network model for prediction of FEV_1.

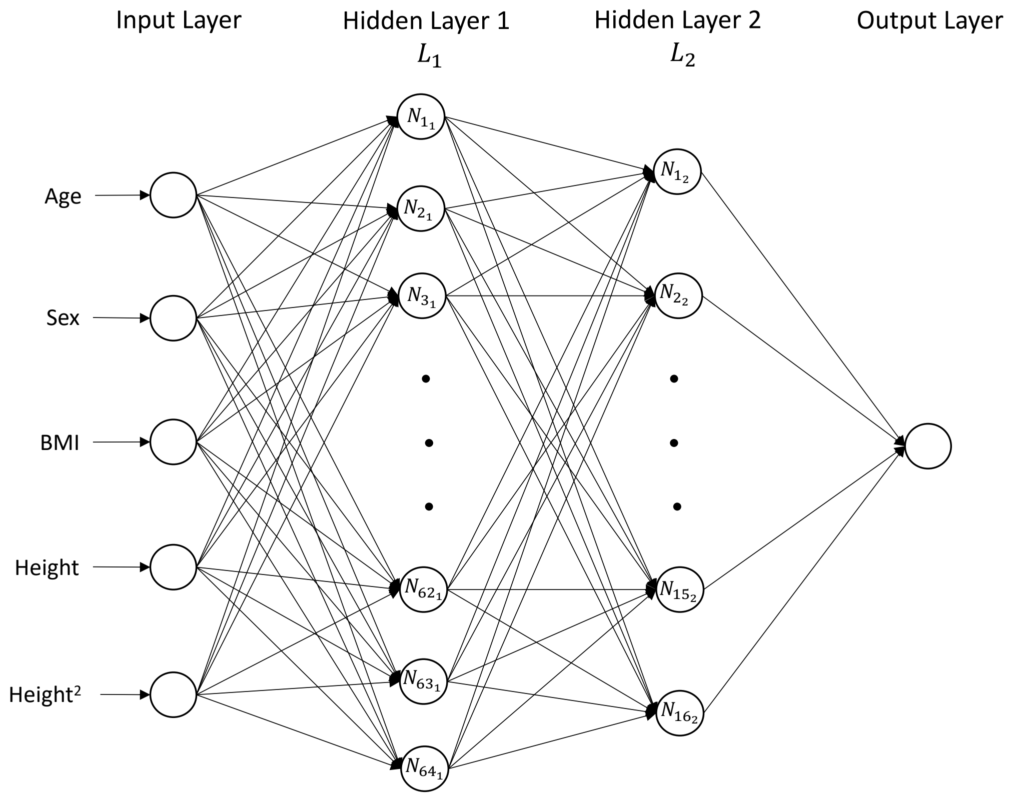

Figure S5: Architecture of neural network model for prediction of smoking cessation and log smoking cessation.

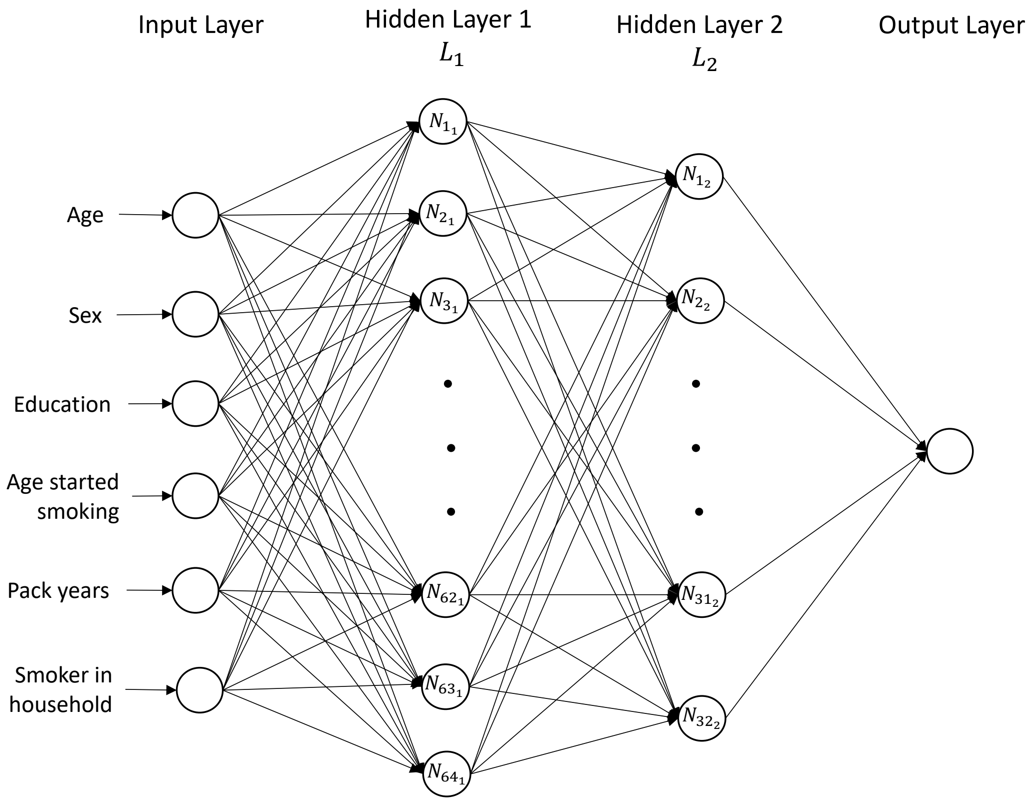

COPDGene Phase 3

Grant Support and Disclaimer

The project described was supported by Award Number U01 HL089897 and Award Number U01 HL089856 from the National Heart, Lung, and Blood Institute. The content is solely the responsibility of the authors and does not necessarily represent the official views of the National Heart, Lung, and Blood Institute or the National Institutes of Health.

COPD Foundation Funding

COPDGene is also supported by the COPD Foundation through contributions made to an Industry Advisory Board that has included AstraZeneca, Bayer Pharmaceuticals, Boehringer-Ingelheim, Genentech, GlaxoSmithKline, Novartis, Pfizer, and Sunovion.

COPDGene® Investigators – Core Units

Administrative Center: James D. Crapo, MD (PI); Edwin K. Silverman, MD, PhD (PI); Barry J. Make, MD; Elizabeth A. Regan, MD, PhD

Genetic Analysis Center: Terri H. Beaty, PhD; Peter J. Castaldi, MD, MSc; Michael H. Cho, MD, MPH; Dawn L. DeMeo, MD, MPH; Adel El Boueiz, MD, MMSc; Marilyn G. Foreman, MD, MS; Auyon Ghosh, MD; Lystra P. Hayden, MD, MMSc; Craig P. Hersh, MD, MPH; Jacqueline Hetmanski, MS; Brian D. Hobbs, MD, MMSc; John E. Hokanson, MPH, PhD; Wonji Kim, PhD; Nan Laird, PhD; Christoph Lange, PhD; Sharon M. Lutz, PhD; Merry-Lynn McDonald, PhD; Dmitry Prokopenko, PhD; Matthew Moll, MD, MPH; Jarrett Morrow, PhD; Dandi Qiao, PhD; Elizabeth A. Regan, MD, PhD; Aabida Saferali, PhD; Phuwanat Sakornsakolpat, MD; Edwin K. Silverman, MD, PhD; Emily S. Wan, MD; Jeong Yun, MD, MPH

Imaging Center: Juan Pablo Centeno; Jean-Paul Charbonnier, PhD; Harvey O. Coxson, PhD; Craig J. Galban, PhD; MeiLan K. Han, MD, MS; Eric A. Hoffman, Stephen Humphries, PhD; Francine L. Jacobson, MD, MPH; Philip F. Judy, PhD; Ella A. Kazerooni, MD; Alex Kluiber; David A. Lynch, MB; Pietro Nardelli, PhD; John D. Newell, Jr., MD; Aleena Notary; Andrea Oh, MD; Elizabeth A. Regan, MD, PhD; James C. Ross, PhD; Raul San Jose Estepar, PhD; Joyce Schroeder, MD; Jered Sieren; Berend C. Stoel, PhD; Juerg Tschirren, PhD; Edwin Van Beek, MD, PhD; Bram van Ginneken, PhD; Eva van Rikxoort, PhD; Gonzalo Vegas Sanchez-Ferrero, PhD; Lucas Veitel; George R. Washko, MD; Carla G. Wilson, MS;

PFT QA Center, Salt Lake City, UT: Robert Jensen, PhD

Data Coordinating Center and Biostatistics, *National Jewish Health, Denver, CO*: Douglas Everett, PhD; Jim Crooks, PhD; Katherine Pratte, PhD; Matt Strand, PhD; Carla G. Wilson, MS

Epidemiology Core, *University of Colorado Anschutz Medical Campus, Aurora, CO*: John E. Hokanson, MPH, PhD; Erin Austin, PhD; Gregory Kinney, MPH, PhD; Sharon M. Lutz, PhD; Kendra A. Young, PhD

Mortality Adjudication Core: Surya P. Bhatt, MD; Jessica Bon, MD; Alejandro A. Diaz, MD, MPH; MeiLan K. Han, MD, MS; Barry Make, MD; Susan Murray, ScD; Elizabeth Regan, MD; Xavier Soler, MD; Carla G. Wilson, MS

Biomarker Core: Russell P. Bowler, MD, PhD; Katerina Kechris, PhD; Farnoush Banaei-Kashani, PhD

COPDGene® Investigators – Clinical Centers

Ann Arbor VA: Jeffrey L. Curtis, MD; Perry G. Pernicano, MD

Baylor College of Medicine, Houston, TX: Nicola Hanania, MD, MS; Mustafa Atik, MD; Aladin Boriek, PhD; Kalpatha Guntupalli, MD; Elizabeth Guy, MD; Amit Parulekar, MD;

Brigham and Women's Hospital, Boston, MA: Dawn L. DeMeo, MD, MPH; Craig Hersh, MD, MPH; Francine L. Jacobson, MD, MPH; George Washko, MD

Columbia University, New York, NY: R. Graham Barr, MD, DrPH; John Austin, MD; Belinda D'Souza, MD; Byron Thomashow, MD

Duke University Medical Center, Durham, NC: Neil MacIntyre, Jr., MD; H. Page McAdams, MD; Lacey Washington, MD

HealthPartners Research Institute, Minneapolis, MN: Charlene McEvoy, MD, MPH; Joseph Tashjian, MD

Johns Hopkins University, Baltimore, MD: Robert Wise, MD; Robert Brown, MD; Nadia N. Hansel, MD, MPH; Karen Horton, MD; Allison Lambert, MD, MHS; Nirupama Putcha, MD, MHS

Lundquist Institute for Biomedical Innovation at Harbor UCLA Medical Center, Torrance, CA: Richard Casaburi, PhD, MD; Alessandra Adami, PhD; Matthew Budoff, MD; Hans Fischer, MD; Janos Porszasz, MD, PhD; Harry Rossiter, PhD; William Stringer, MD

Michael E. DeBakey VAMC, Houston, *TX*: Amir Sharafkhaneh, MD, PhD; Charlie Lan, DO

Minneapolis VA: Christine Wendt, MD; Brian Bell, MD; Ken M. Kunisaki, MD, MS

Morehouse School of Medicine, Atlanta, GA: Eric L. Flenaugh, MD; Hirut Gebrekristos, PhD; Mario Ponce, MD; Silanath Terpenning, MD; Gloria Westney, MD, MS

National Jewish Health, Denver, CO: Russell Bowler, MD, PhD; David A. Lynch, MB

Reliant Medical Group, Worcester, MA: Richard Rosiello, MD; David Pace, MD

Temple University, Philadelphia, PA: Gerard Criner, MD; David Ciccolella, MD; Francis Cordova, MD; Chandra Dass, MD; Gilbert D'Alonzo, DO; Parag Desai, MD; Michael Jacobs, PharmD; Steven Kelsen, MD, PhD; Victor Kim, MD; A. James Mamary, MD; Nathaniel

Marchetti, DO; Aditi Satti, MD; Kartik Shenoy, MD; Robert M. Steiner, MD; Alex Swift, MD; Irene Swift, MD; Maria Elena Vega-Sanchez, MD

University of Alabama, Birmingham, AL: Mark Dransfield, MD; William Bailey, MD; Surya P. Bhatt, MD; Anand Iyer, MD; Hrudaya Nath, MD; J. Michael Wells, MD

University of California, San Diego, CA: Douglas Conrad, MD; Xavier Soler, MD, PhD; Andrew Yen, MD

University of Iowa, Iowa City, IA: Alejandro P. Comellas, MD; Karin F. Hoth, PhD; John Newell, Jr., MD; Brad Thompson, MD

University of Michigan, Ann Arbor, MI: MeiLan K. Han, MD MS; Ella Kazerooni, MD MS; Wassim Labaki, MD MS; Craig Galban, PhD; Dharshan Vummidi, MD

University of Minnesota, Minneapolis, MN: Joanne Billings, MD; Abbie Begnaud, MD; Tadashi Allen, MD

University of Pittsburgh, Pittsburgh, PA: Frank Sciurba, MD; Jessica Bon, MD; Divay Chandra, MD, MSc; Joel Weissfeld, MD, MPH

University of Texas Health, San Antonio, San Antonio, TX: Antonio Anzueto, MD; Sandra Adams, MD; Diego Maselli-Caceres, MD; Mario E. Ruiz, MD; Harjinder Singh
